# Lead poisoning in children: a case report

**DOI:** 10.11604/pamj.2016.24.316.10352

**Published:** 2016-08-18

**Authors:** Zineb Jouhadi, Dalal Bensabbahia, Fouad Chafiq, Bouchra Oukkache, Nisrine Bennani Guebessi, El Abidi Abdellah, Jilali Najib

**Affiliations:** 1 Pediatric Infectious Diseases Children’s Hospital, Casablanca, Morocco; 2 Poison Control Center and Pharmaco-vigilance, Rabat, Morocco; 3 Hematology Laboratory, Casablanca, Morocco; 4 Department of Pathology, Casablanca, Morocco; 5 Department of Toxicology, National Institute of Hygiene, Rabat, Medical school, university Hassan II, Casablanca, Morocco

**Keywords:** Lead poisoning, lead colic, children

## Abstract

Lead colic is a rare cause of abdominal pain. The diagnosis of lead poisoning is most often mentioned in at risk populations (children, psychotic). We report the case of a 2 year old child that was presented for acute abdomen. Abdominal plain radiograph showed multiple intra-colonic metallic particles and suggested lead poisoning diagnosis. Anamnesis found a notion of pica and consumption of peeling paint. Elevated blood lead levels (BLL) confirmed the diagnosis. The lead poisoning is a public health problem especially in children, but its manifestation by a lead colic is rare and could simulate an acute abdomen table.

## Introduction

Lead poisoning (LP) is a potentially serious illness. It may be revealed by a pseudo-surgical table “lead colic" with abdominal pain and abdominal defense. The diagnosis is most often raised in a context of professional exposure or, more rarely, in populations at risk of contact with lead especially in children under six years due to their oral behavior and unhealthy housing. The aim of our work is to sensitize practitioners to the problem of lead poisoning in general and draw their attention to this special and very rare manifestation that is lead colic.

## Patient and observation

**Figure 1:**
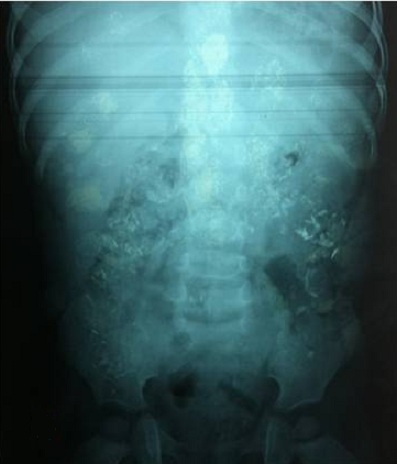
Abdomen X-ray: irregular punctate opacities mainly colic seat

**Figure 2:**
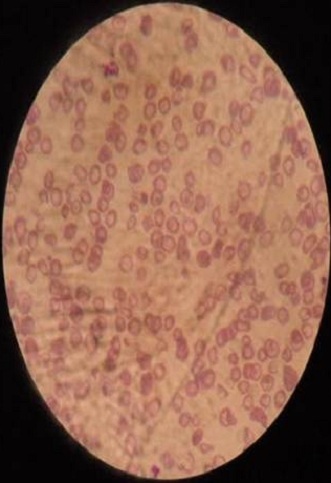
Blood smear: basophilic inclusions in erythrocytes

**Figure 3:**
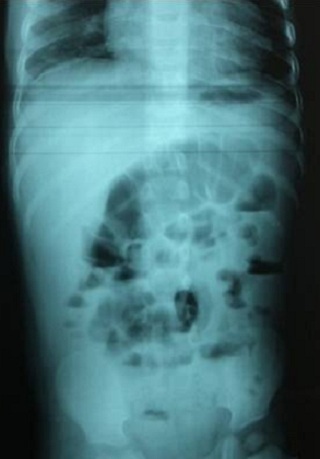
Abdomen X-ray: disappearance of metallic opacities 48 hours after a laxative treatment

A male infant aged 2 years old, was brought to the pediatric emergency department in an acute abdomen table with vivid abdominal pain associated with vomiting and severe constipation but without real interruption of bowel movement. Clinical examination revealed an infant 11 kg, tired, pale but with good hemodynamic constants, no dehydrated, no fever (T: 36.4°). The physical examination of the abdomen showed widespread defense and sensitivity without contracture, the remaining of the physical examination was normal. A plain X-ray of the abdomen showed irregular punctate opacities mainly colic seat (Figure 1). Abdominal ultrasound showed no abnormalities; a surgical emergency was then discarded. The child was referred to our medical facility for additional support Anamnestic investigations revealed a notion of pica with ingestion of flaking paint and pencil leads; knowing that the child is living in an old home built in 1960 and repainted in 2009. No consumption of traditional medicine products or use of "kohl" by the baby was found. Further exploration revealed hypochromic microcytic anemia (7.5g/dl; normal 11.0-14.5g/dl); a blood smear revealed the presence of basophilic inclusions in erythrocytes (Figure 2); the liver and kidney function were normal; elevated BLL to 22.8 µg/dl and the rate of vitamin D3 was low at 28ng/l. Examination of the eye fundus did not show greyish studded macula aspect called Sonkin seedlings specific of this intoxication. There was no clear metaphyseal bands on tibial radiographies. Determination of lead in potable water used by this family was within normal limits at 8 µg/dl; the same test was done on paint chips removed from his home that showed a rate of 40 µg/g. Treatment with lactulose-based laxative lifted the constipation and allowed disappearance of abdominal pain. Diarrhea debacle cleaned the colon from its metal content (Figure 3). Chelation therapy was not indicated as the blood lead level was not too high.

## Discussion

Lead poisoning (LP), acute or chronic, professional or domestic is a serious disease that can affect all mammals, birds (avian LP) humans and particularly children [1, 2]. WHO classifies lead among the ten chemicals of concern for public health. Each year, lead exposure causes an estimated 600,000 new cases of mental retardation in children. It would cause 143,000 deaths per year, mostly in developing regions [3]. Lead represents about 0.6% of the global disease burden; about half in the WHO Region of Southeast Asia, a fifth in the Western Pacific, and a fifth on the Eastern Mediterranean. The absorbed lead is carried by the blood. More than 90% is set on the red cells under non-diffusible form. The diffusible fraction of plasma lead (less than 10%) is distributed between the vascular area, tissues (kidney, brain, liver, bone marrow..) and bone area. only 70% (versus 94% in adults) of absorbed lead is deposited in the bones in children, which may explain in part the greater susceptibility of children to the toxic effects of lead. This also means that blood lead levels or BLL (blood lead level) is not an accurate reflect of the total body burden of lead [1, 2]. The half-life in the vascular sector is about 40 days versus several years in bone area, it is even longer in pregnant women and children. Young children tend to put their fingers and objects in their mouths (lead has a sweet taste), some children have compulsive eating disorders that involve ingesting systematically some inedible substances (pica); this is the case of our patient. Digestive absorption (50% versus 10% in adults) and penetration through the skin of lead particles is higher in children. Levels of circulating lead are more important if the child is young [3]. Lead undergoes trans-placental transfer explaining the risk of fetal intoxication when the mother is exposed. The most important passage of metal through the blood-brain barrier in children reflects the predominance of encephalic symptoms in the LP of children. The respiratory tract is the second way of possible contamination (lead vapors). The main sources of lead exposure are old and dilapidated housing, leaded paints are currently prohibited. The paint is ingested in the form of scales and inhaled as dust produced during degradation over time or during work (façade renovation, scraping, brushing, sanding) [4]. A large part of lead toxicity results from it’s binding to calcium-activated protein with much higher affinity and thereby interferes with different calcium-dependent cell functions such as heme production, it thus results in hypochromic microcytic anemia as is the case of our patient with anemia of 7.5 g/dl. In the kidney, lead causes an alteration of the tubular function manifested by aciduria, glycosuria and hyper-phosphaturia. Chronic exposure to lead causes a reversible nephritis and alters the synthesis of vitamin D. Indeed, our patient had a vitamin D deficiency [1, 2].

Pediatric neurological involvement is more frequent and more severe. At high levels of exposure, lead affects the central nervous system, causing coma, convulsions and death. Children who survive to acute lead poisoning or who are exposed to chronic poisoning may suffer from disabling and irreversible consequences such as behavioral and cognitive impairment that affects school performance; indeed, meta-analyzes have shown that an elevation of 100 micrograms / l resulted in a decreased 1 to 3 points of Intelligence Quotient (IQ) [5]. The blood dyscrasias manifested by often microcytic hypochromic anemia because often associated with iron deficiency, which explains the weakness in these children. An hemolytic anemia could be seen with high BLL value (> 70 µg/dl) [6]. Abdominal manifestations of lead poisoning in its typical form is lead colic; very vivid pains in the abdomen radiating from the umbilicus toward the lumbar regions, the thighs and the testicles, associated with obstinate constipation and sometimes vomiting, no fever, non-abdominal contracture without radiographic evidence of pneumo-peritoneum or occlusion, abdomen compression above the navel relieves the pain. Nowadays, it is rare to observe lead colic, which is common when BLL exceeds 10,6 g/dl. Intoxicated patients with BLL greater than 50,6 g/dL will often complain of vague abdominal pain and constipation [6]. BLL reflects the current status of the balance between absorbed Lead and lead stored in the tissues. The presence of lead in the blood must not be tolerated, because even minimal blood lead levels have been associated with subtle neurobehavioral deficits and most children with lead poisoning are asymptomatic [5] in this regard CDC(Centers for Disease Control ) fixed in 2012the reference threshold value of blood lead levels in 5 µg / dl [7]. BLL must be supplemented by laboratory tests in search of stigma of lead poisoning including a blood count that objective hemoglobin decreased and blood smear showing basophilic granules of red blood cells.

Treatment of lead poisoning consists in the identification of the sources of lead and their eradication whenever possible, lifestyle changes, symptomatic treatment; decontamination and special treatment are sometimes needed [8]. Symptomatic treatment includes hospitalization in ICU with ventilator support if cerebral edema; antispasmodic to treat lead colic, anti-comitial therapy in case of encephalopathy, dialysis in case of severe kidney disease, zinc intake reduces the toxicity of lead. Decontamination processing in cases of acute intoxication by mouth is achieved by gastric lavage with a solution precipitating lead as insoluble lead sulfate (40 g of sodium and magnesium sulfate per1litre of water). A laxative treatment is recommended in the presence of intestinal opacity [8]. The specific chelation therapy is achieved by substances that can attach the metal component and become a soluble and non-toxic compound eliminable in urine. In children, it is recommended according to the CDC from a BLL at 45µg/dL [7]. Before starting treatment, a blood test is required for confirmation, hospitalization is required and the type of chelation therapy is recommended depending on the concentration of lead levels and the clinical picture. There are 3 types of chelating agents: calcium EDTA (ethylene diamine-tetra-acetate) DMSA (2, 3-dimercaptosuccinic acid succimer) succicaptal^®^ and BAL (dimercaptoprol). The chelator available in Morocco is DMSA (succicaptal^®^) [7–9]. It is an alternative increasingly interesting in the treatment of lead poisoning. It mobilizes the active metabolic lead soft tissue and would not lead to elevation of intra-cerebral lead or depletion of essential elements (Fe, Ca, Mg, Zn). It’s used orally at a dose of 30 mg/kg/day, three times daily during 5 days then at 10 mg/kg/ 12 hours for 2 weeks. The maximum daily dose is 1.8 g for adults [10]. We have not used the chelation therapy in the absence of clinical signs of severity and blood BLL at 22.8 µg/dl (threshold for chelation is greater than 45 µg/dl). The monitoring depends on the initial BLL and age of the child.

## Conclusion

Lead poisoning is not an enough known pathology by practitioners. It can affect the mental future of our children and lead to neurological or kidney damages. The remedy of this poisoning can only be preventive. Ignorance of lead poisoning can lead to serious complications, diagnostic mistakes, aggressive and expensive exams. Once evoked; its confirmation is easy by a BLL which is correlated to the toxic effects of lead. Prevention of LP must be part of the national health-environment protection plan. It relies on outreach and public information. The general practitioner or pediatrician aware of this condition has a major role in the functioning of the national surveillance and reporting of cases of childhood LP so this affection should benefit from a specific instruction in initial and continuing medical education for practitioners.
